# Imidazolium Ionic Liquids as Corrosion Inhibitors: Insights into Film Impermeability to Corrosive Species

**DOI:** 10.3390/molecules30224383

**Published:** 2025-11-13

**Authors:** Ruizhao Ye, Yibo Zhang, Chi-Min Shu, Juncheng Pu, Chongwei An, Fusheng Cui

**Affiliations:** 1 School of Environmental and Safety Engineering, North University of China, Taiyuan 030051, China; yrz051110@163.com (R.Y.); zyb18306839978@163.com (Y.Z.); pjc7777777@163.com (J.P.); anchongwei@yeah.net (C.A.); 2 Center for Process Safety and Industrial Disaster Prevention, School of Engineering, National Yunlin University of Science and Technology, Yunlin 64002, Taiwan; shucm@yuntech.edu.tw

**Keywords:** ionic liquid corrosion inhibitors, permeability resistance, surface roughness, confocal microscopy, electrochemical impedance spectroscopy

## Abstract

The quantitative evaluation of permeability resistance remains a major challenge in the assessment of IL corrosion inhibitors. Here, we presented a morphology-based methodology that combined electrochemical impedance spectroscopy for inhibition coverage with confocal microscopy three-dimensional analysis to quantify surface roughness (S_a_), thereby establishing a dual-criterion framework. At high inhibition efficiency (>73%), surface roughness ranking at identical concentrations directly reflected permeability resistance, whereas under insufficient efficiency, concentration-gradient experiments effectively eliminated coverage interference. Application to three chemically different imidazolium-based ILs ([C_3_mim][OTf], [C_3_mim][NO_3_], and [C_3_mim][Br]), which were studied at three different concentrations (10, 30, and 50 mM), revealed a nonlinear relationship between inhibition efficiency and surface roughness, with the nitrate system exhibiting the most favorable permeability resistance. This strategy provided a critical dimension for the quantitative evaluation of IL corrosion inhibitors and advanced the understanding of their protective mechanisms.

## 1. Introduction

Corrosion protection of metallic materials represents a critical challenge for the sustainable development of modern industry, particularly in harsh environments such as petrochemical processing and marine engineering. Owing to their cost-effectiveness and operational convenience, corrosion inhibitors have become the core component of protective systems [1]. In recent years, ionic liquids (ILs) have attracted considerable attention in the field of green corrosion inhibitors due to their tunable molecular structures, negligible vapor pressure, high thermal stability, and unique solubility characteristics, making them highly promising candidates [2,3,4,5,6]. Studies have demonstrated that by tailoring different cation–anion combinations, it is possible to construct more than 10^19^ ILs. Certain ILs with specific structures can adsorb onto diverse metallic surfaces to form interfacial inhibitors with high efficiency [7]. This occurs because the synergistic interaction between cations and metals enables these ionic liquids to form dense, firmly adherent, and stable protective films on various metallic substrates. For instance, the inhibition performance of imidazolium-based inhibitors depends on the specific interactions between their functional groups and the metal surface. This is attributed to the presence of the –C = N– functional group and the electronegative nitrogen atom within the molecular structure, which facilitate strong coordination and adsorption on the metal surface, thereby providing excellent corrosion resistance in aggressive environments [8]. Therefore, systematic investigation into the performance of ILs is of pronounced importance.

As early as 2009, Zhang et al. reported that 1-Butyl-3-methylimidazolium chloride and 1-Butyl-3-methylimidazolium hydrogen sulfate could inhibit the acid corrosion of low-carbon steel in acidic environments [8]. In addition, in 2021, Xue et al. investigated the corrosion inhibition behavior of pyridinium-based ILs with different alkyl chain lengths on N80 steel in hydrochloric acid solution and found that N-octyl-4-methylpyridinium bromide exhibited the highest inhibition efficiency, exceeding 90% [9]. More recently, Hu et al. from China University of Petroleum (East China) reported two novel imidazolium-based ILs inhibitors—1-benzylethyl-3-(3-phenylpropyl) imidazolium hexafluorophosphate (PPIPF_6_) and 1,3-diphenylimidazolium hexafluorophosphate (DPIPF_6_)—and evaluated their anticorrosion performance on carbon steel in 1 M HCl (1 molar hydrochloric acid). The results showed that at an ILs concentration of 1 × 10^−3^ M, the inhibition efficiencies of PPIPF_6_ and DPIPF_6_ reached 94.8% and 94.4%, respectively [10]. Furthermore, Li et al. combined molecular dynamics simulations with density functional theory to elucidate the adsorption configurations of ILs at metal interfaces. Their findings indicated that long-chain alkyl imidazolium ILs could repel corrosive species, such as Cl^−^ via electrostatic interactions and lessen the contact between the metal surface and water molecules [11]. All-atom molecular dynamics (MD) simulations can provide deep insights into the coordination modes, hydrogen-bond interactions, and adsorption configurations of ions at metal or oxide interfaces, thereby bridging the gap between microscopic interactions and macroscopic inhibition performance. For example, Stanković et al. employed all-atom MD simulations to investigate the effects of water content on the transport and thermodynamic properties of phosphonium ionic liquids, revealing the critical role of water–ion interactions in regulating viscosity and ionic mobility [12]. At larger spatial scales, coarse-grained molecular dynamics (CG-MD) simulations can be used to explore the flow and lubrication behaviors of ionic liquids under confinement. Dašić et al. found that nanoscale confinement significantly alters the structural layering and shear response of ILs [13]. These simulation studies complement experimental findings and provide molecular-level support for a deeper understanding of the corrosion inhibition mechanisms of ILs.

Although the above studies have made notable progress, research on ILs still primarily follows the traditional paradigm of assessing surface coverage and inhibition efficiency. In contrast, systematic investigations into another key protective property—the resistance of ILs films to the permeation of corrosive species—remain largely absent. Owing to the bulky molecular structures of ILs and the complex interactions between cations and anions, their interfacial films exhibit a high degree of structural tunability, offering unique molecular design space to optimize film compactness. However, the structure–property relationship governing this tunability is not yet fully understood. As industrial environments become increasingly complex (e.g., high flow rates, multi-ion coexistence, extreme temperature/pressure), the limitations of the coverage-based theory have become more pronounced: high coverage (even >95%) does not always guarantee long-term or reliable protection. Fundamentally, coverage is a static descriptor that reflects the overall inhibition efficiency—namely, the fraction of the metal surface occupied by the protective film—yet it does not adequately capture the film’s ability to dynamically resist penetration by corrosive media, particularly aggressive ions, such as Cl^−^. We hypothesize that, under practical conditions of high ionic strength and hydrodynamic impact, the molecular packing density, structural integrity, interfacial bonding strength, and mechanical stability of the ILs film—collectively defining its film impermeability to corrosive species (hereafter referred to as anti-permeability)—represent the decisive dynamic performance that governs whether the protective film can effectively block corrosive species.

Thus, it is the unification of coverage and anti-permeability that provides a more comprehensive criterion for evaluating the effectiveness of ILs. The lack of in-depth understanding and precise evaluation of anti-permeability has become a major bottleneck restricting the optimization and engineering applicability of corrosion inhibitors. Current assessment methods—such as relying on single impedance modulus or contact angle measurements—are indirect and cannot directly or quantitatively reveal internal structural defects or permeation pathways within the film. Extending from prior studies that evaluated compactness through surface morphology analysis [14], it is reasonable to infer that a smoother post-corrosion surface indicates a denser ILs film and better overall inhibition efficiency. However, if the ranking of inhibition efficiency does not align with the trends in surface roughness, this discrepancy suggests the independent existence of anti-permeability as a distinct property. Together with coverage, it jointly determines the protective performance of ILs, while surface roughness can serve as an effective metric for evaluating anti-permeability.

Based on this rationale, our study employed electrochemical measurements to characterize overall inhibition efficiency (coverage), coupled with three-dimensional surface roughness quantification using confocal microscopy. We investigated how three imidazolium-based ILs inhibitors, at varying concentrations, affected the surface roughness of corroded metal specimens and the corresponding inhibition performance, thereby elucidating the anti-permeability characteristics of different ILs.

## 2. Results and Discussion

### 2.1. EIS Measurements

Electrochemical testing was conducted to verify the overall corrosion resistance of ILs inhibitors against external corrosive media, which reflected their surface coverage on Q235 electrodes (Carbon structural steel, working electrode) and served as the criterion for evaluating overall inhibition performance. EIS measurements were performed to investigate the inhibition behavior of Q235 working electrodes in the presence of three different ILs (1 cation with 3 different anions)—[C_3_mim][OTf], [C_3_mim][NO_3_], and [C_3_mim][Br]—at concentrations of 10, 30, and 50 mM.

For concentration selection, 10 mM was initially adopted as the starting point to ensure the ionic liquid system possessed fundamental corrosion inhibition capability. Experimental results indicated that overall corrosion inhibition effectiveness was limited at low concentrations, prompting gradual increases to 30 mM and 50 mM. Findings demonstrated that within this concentration range, the trend in anti-permeation performance of the ionic liquid became distinctly evident, sufficiently reflecting the influence of concentration on system performance. Consequently, no further concentration increases were pursued.

**Figure 1:**
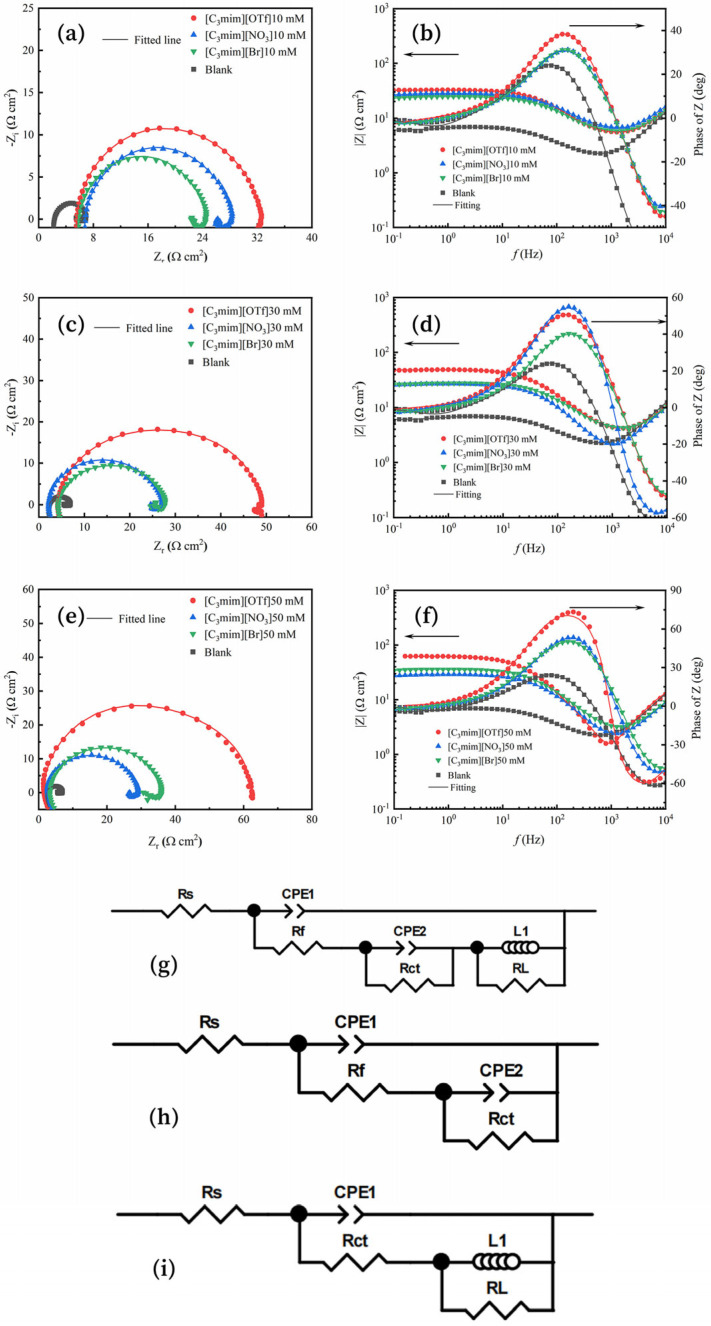
(**a**,**c**,**e**) Nyquist plots and (**b**,**d**,**f**) Bode plots for Q235 steel in test solutions containing different concentrations (0, 10, 30, 50 mM) of [C_3_mim][OTf], [C_3_mim][NO_3_], and [C_3_mim][Br]. Equivalent electrical circuits used to fit the EIS data for the ILs: (**i**) in blank solution (no inhibitor), and (**g**,**h**) in inhibitor-containing solution, where (**h**) represents the equivalent circuit excluding inductance for 50 mM [C_3_mim][OTf].

The Nyquist and Bode plots of Q235 in solutions containing these ILs at different concentrations (0, 10, 30, and 50 mM) are shown in Figure 1a–f. Meanwhile, the equivalent electrical circuits used to fit the Electrochemical Impedance Spectroscopy (EIS) data are presented in Figure 1g–i. For the case of 50 mM [C_3_mim][OTf], no inductive component was observed; therefore, the circuit shown in Figure 1h was adopted as the corresponding equivalent model. The electrochemical impedance spectroscopy (EIS) data were fitted using ZSimpWin (3.6) software, and the fitting quality was evaluated by the chi-squared (χ^2^) value (see Table 1). All obtained χ^2^ values were in the range of 10^−3^–10^−5^, indicating high fitting quality and good agreement between the experimental spectra and the simulated curves.

As shown in Figure 1a,c,e, each spectrum consists of a high-frequency capacitive loop and a low-frequency inductive loop. In the uninhibited solution, the capacitive loop was typically attributed to the charge-transfer resistance (R_ct_), whereas in the inhibited solutions it arose from both R_ct_ and the film resistance (R_f_) [15]. The inductive loop was generally associated with the relaxation process of adsorbed species [16]. These findings indicate that the corrosion of Q235 in the test solutions is predominantly controlled by charge transfer [17]. Upon the addition of ILs, the diameters of the capacitive loops increase, demonstrating that all three ILs exhibit pronounced inhibition effects on Q235 corrosion in the H_2_S–HCl–H_2_O system. Furthermore, the diameter of the high-frequency capacitive loops increased with rising ILs concentration (Figure 1b,d,f), while the low-frequency impedance modulus and phase angle also increased correspondingly. These results suggested that, as interfacial inhibitors, the effectiveness of the three ILs is further enhanced with increasing concentration [7,18].

As illustrated in Figure 1g–i, R_s_ represents the solution resistance, R_f_ denotes the film resistance, L and R_L_ corresponded to the inductive elements, CPE_1_ was the constant phase element associated with the film, and CPE_2_ was the constant phase element related to the double layer [15]. The inhibition efficiency (η) was used to evaluate the overall corrosion performance of different ILs, and was calculated according to Equation (1), where R_p,o_ and R_p,i_ represent the polarization resistance in the absence and presence of the ILs inhibitor, respectively. The polarization resistance R_p_ in the equivalent circuit (Figure 1i) was calculated using Equation (2), The polarization resistance (R_p_) in the equivalent circuit (Figure 1g) was calculated using Equation (3), For the case of 50 mM trifluoromethanesulfonate, no inductive element was observed; therefore, the corresponding equivalent circuit is shown in (Figure 1h), and the polarization resistance (R_p_) was calculated according to Equation (4) [19]:

(1) $$\eta =\frac{R_{p,i}-R_{p,0}}{R_{p,i}}\times 100$$

(2) $$R_{p}=R_{ct}+R_{L}$$

(3) $$R_{p}=R_{ct}+R_{L}$$

(4) $$R_{p}{=R}_{f}+R_{ct}$$

As shown in Table 1, the EIS parameters of Q235 obtained from the electrochemical equivalent circuits confirmed that all three ILs increased R_p_, thereby suppressing corrosion. Among them, [C_3_mim][OTf] consistently exhibited the highest efficiency, outperforming [C_3_mim][NO_3_] and [C_3_mim][Br] across all tested concentrations. However, at 50 mM, [C_3_mim][Br] surpassed [C_3_mim][NO_3_] due to a more pronounced increase in inhibition efficiency, underscoring the distinct concentration-dependent behaviors of the three inhibitors. This divergence is most likely attributable to differences in anion adsorption strength and film compactness, which together governed both the coverage and anti-permeability of the protective films.

### 2.2. Surface Roughness Evaluation of Corroded Specimens

#### 2.2.1. Surface Morphology Analysis

**Figure 2:**
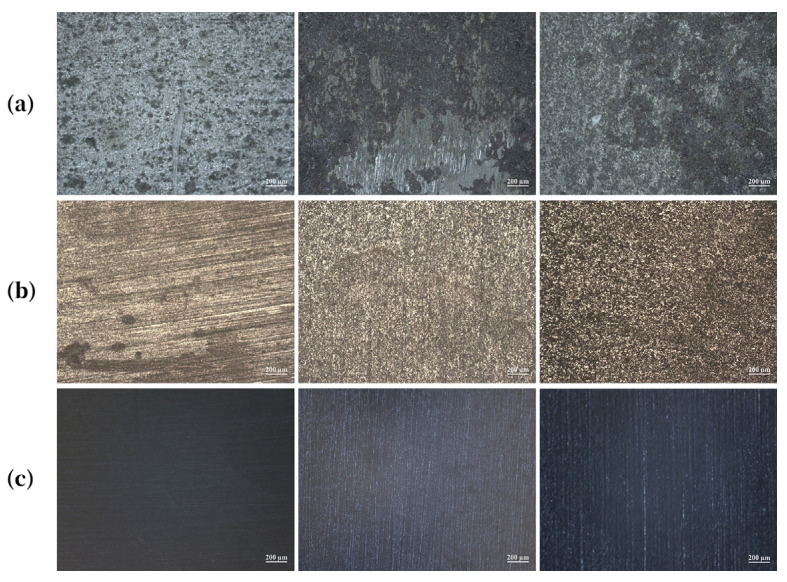
Representative SEM images of Q235 steel specimen surfaces after exposure to solutions containing (**a**) 10 mM, (**b**) 30 mM, and (**c**) 50 mM concentrations of the ionic liquids; from left to right: [C_3_mim][NO_3_], [C_3_mim][OTf], and [C_3_mim][Br].

As shown in Figure 2, at the same concentration, the surface of Q235 specimens exposed to [C_3_mim][NO_3_] appeared visibly smoother, with the polishing marks from sample preparation largely preserved. In comparison, the surface treated with [C_3_mim][OTf] was less smooth, while that treated with [C_3_mim][Br] exhibited the roughest morphology, characterized by numerous corrosion pits. This indicated that the bromide provides weaker protection, forming a less compact IL film, whereas the nitrate delivered the strongest protection, even outperforming the trifluoromethanesulfonate. Notably, this deviates from the overall inhibition efficiency trend, suggesting that nitrate exhibited superior anti-permeability performance. Furthermore, comparison across concentrations revealed that, for each IL, the specimen surface became progressively smoother with increasing concentration. This demonstrated that higher IL concentrations strengthened the integrity of the protective film and ameliorated its ability to resist corrosive penetration.

#### 2.2.2. Arithmetic Mean Surface Roughness Analysis

As shown in Table 2, at identical ILs concentrations, the surface roughness consistently followed the ascending order: [C_3_mim][NO_3_] < [C_3_mim][OTf] < [C_3_mim][Br]. These results indicated that the nitrate forms the most compact film, providing the most effective protection for Q235 steel. For each IL, increasing the concentration led to progressively lower surface roughness values, suggesting that higher concentrations enhance the anti-permeability of the ILs films against corrosive media.

Moreover, the variation rates of inhibition efficiency and surface roughness exhibited consistent trends with concentration. Taking the nitrate as an example, both parameters initially increased expeditiously with concentration before reaching a more gradual regime. This finding highlighted the importance of maintaining sufficiently high surface coverage when evaluating anti-permeability: only when the metal surface is extensively covered can the influence of rougher uncovered regions be minimized, thereby allowing surface roughness to serve as an effective indicator of anti-permeability.

#### 2.2.3. Three-Dimensional Surface Morphology Analysis

**Figure 3:**
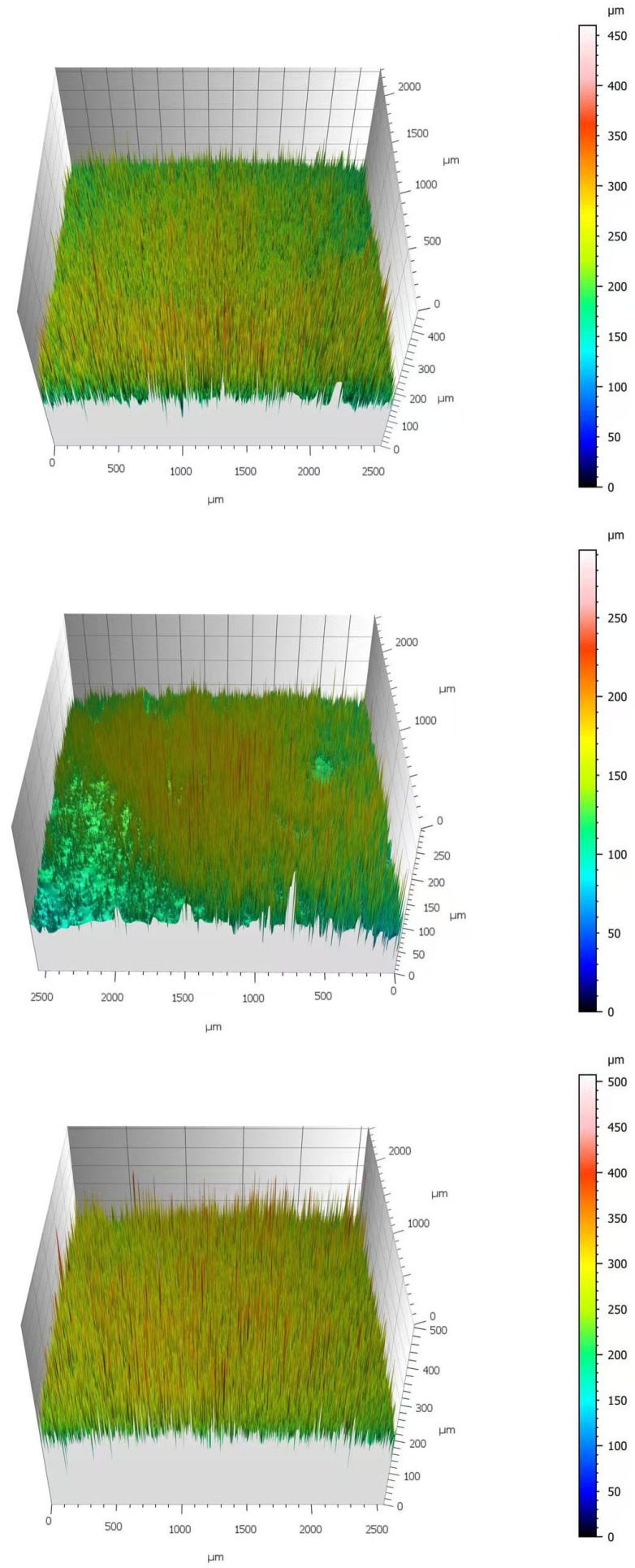
Comparative 3D surface topography images of Q235 steel exposed to 30 mM solutions: (from top to bottom) [C_3_mim][OTf], [C_3_mim][NO_3_], and [C_3_mim][Br].

As shown in Figure 3, the three-dimensional (3D) surface topographies exhibited consistent trends at identical ionic liquid concentrations. Taking the 30 mM condition as an example, the specimen treated with [C_3_mim][Br] displayed the highest color scale threshold, exceeding 500 μm. Its 3D image appeared highly irregular and visually rough, indicating a low film compactness and poor resistance to corrosive penetration. In contrast, the specimen treated with [C_3_mim][NO_3_] exhibited the smallest color scale range (approximately 0–250 μm) and a relatively smooth surface, confirming that this IL formed a denser protective film with superior protective capacity. Furthermore, increasing IL concentration consistently abated surface roughness across all three systems, in full agreement with the arithmetic mean roughness (S_a_) values obtained from software analysis (Confomap).

#### 2.2.4. Cross-Sectional Profile Analysis

**Figure 4:**
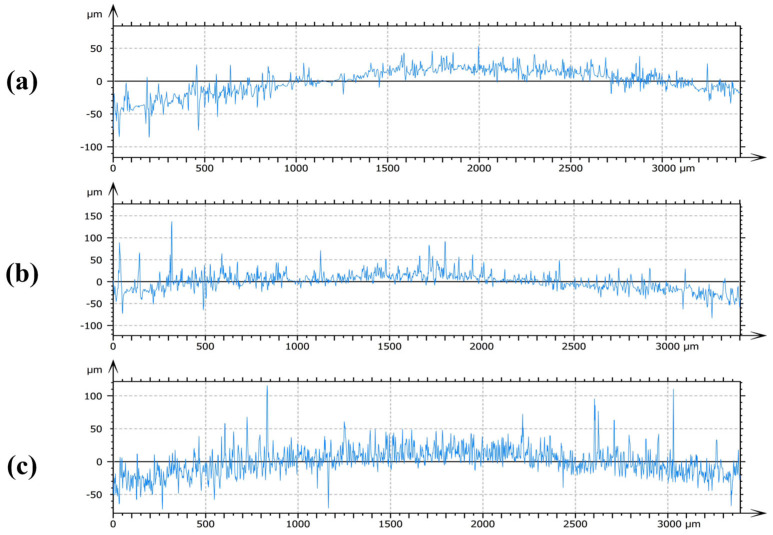
Presents representative cross-sectional profiles of Q235 steel specimens exposed to 30 mM solutions of the ionic liquids: (**a**) 1-propyl-3-methylimidazolium nitrate ([C_3_mim][NO_3_]), (**b**) 1-propyl-3-methylimidazolium trifluoromethanesulfonate ([C_3_mim][OTf]), and (**c**) 1-propyl-3-methylimidazolium bromide ([C_3_mim][Br]).

In correspondence with the 3D surface views in Figure 3, cross-sectional profiles were analyzed along the diagonals of the corroded specimens, with 30 mM as a representative case. As shown in Figure 4, the profile of the specimen treated with [C_3_mim][NO_3_] (Figure 4a) fluctuated narrowly within the −50 to 50 μm range, indicating effective surface protection. In contrast, the specimen treated with [C_3_mim][OTf] (Figure 4b) fluctuated mainly within −50 to 100 μm, with more frequent and larger oscillations compared with the nitrate. The bromide-treated specimen (Figure 4c) also fluctuated within −50 to 100 μm but exhibited highly irregular, high-frequency oscillations, suggesting inferior film quality and weaker protective ability. Integrating these observations with the 3D surface analyses provided a comprehensive evaluation of the surface roughness of corroded specimens.

### 2.3. Anti-Permeability Evaluation of Inhibitors

By comparing the rankings derived from overall inhibition efficiency and surface roughness, noticeable deviations were observed. Specifically, under conditions of high inhibition efficiency, the roughness ranking diverged from the efficiency trend. For example, [C_3_mim][OTf] exhibited the highest inhibition efficiency but a poorer surface roughness compared with [C_3_mim][NO_3_]. This indicated that higher inhibition efficiency did not necessarily correspond to smoother surfaces, and that coverage, as reflected by overall efficiency, was not directly linked to film compactness.

In contrast, superior surface roughness implies curtailed penetration of corrosive species, highlighting the stronger anti-permeability of the protective film. These findings suggested that anti-permeability and inhibition efficiency represent two complementary properties that jointly determined the effectiveness of IL inhibitors. Moreover, surface roughness served as a reliable indicator for evaluating anti-permeability, as it reflected differences in film compactness.

### 2.4. Mechanistic Discussion on Film Densification

The superior compactness of the protective film formed by [C_3_mim][NO_3_] can be attributed to its strong interactions with the imidazolium cation. The nitrate anion, as a moderately coordinating species, forms strong hydrogen-bonding and electrostatic interactions through its oxygen atoms with the C_2_–H and C_4_/C_5_–H sites on the imidazolium ring, promoting an ordered ionic arrangement, enhanced interfacial adhesion, and the formation of a dense, continuous three-dimensional network [20]. In contrast, the trifluoromethanesulfonate ([OTf]^−^) and bromide ([Br]^−^) anions lack exposed oxygen atoms capable of forming strong hydrogen bonds with the imidazolium ring. The bulky [OTf]^−^ anion introduces steric hindrance that disrupts ionic packing, while Br^−^ exhibits relatively weak coordination ability, leading to more loosely packed and defect-prone films. As emphasized by Silva et al. [21], the type of anion determines the strength of the hydrogen-bonding network and the molecular packing between cations; anions containing exposed oxygen atoms and possessing strong coordinating ability (such as [NO_3_]^−^) facilitate the formation of a more compact molecular arrangement. Consequently, the nitrate system demonstrates superior anti-permeability owing to its stronger coordination ability and optimized cation–anion interactions, which collectively yield tighter molecular packing and improved structural integrity of the protective film.

## 3. Materials and Methods

### 3.1. Materials

**Figure 5:**
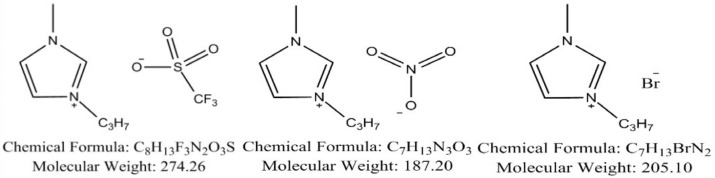
Chemical structures of the studied ionic liquids, from left to right: [C_3_mim][OTf], [C_3_mim][NO_3_], and [C_3_mim][Br].

**IL Preparation.** Three ILs (1 cation with 3 different anions),1-propyl-3-methylimidazolium trifluoromethanesulfonate([C_3_mim][OTf]), 1-propyl-3-methylimidazolium nitrate([C_3_mim][NO_3_]), and 1-propyl-3-methylimidazolium bromide([C_3_mim][Br]), were purchased from Taiyuan Milestone Co., Ltd., (Taiyuan, Shanxi, China) with a stated purity of ≥98%. The molecular structure is shown in Figure 5. The selection of these three ILs—[C_3_mim][OTf], [C_3_mim][NO_3_], and [C_3_mim][Br]—primarily aims to investigate how anion type influences ionic liquid permeability resistance, laying the groundwork for subsequent mechanism analysis. Preliminary literature review suggests these three anions may exhibit significant differences in permeability resistance.

**Preparation of Q235 samples**. Q235 steel specimens were machined into working electrodes for electrochemical impedance spectroscopy (EIS) measurements, with dimensions of 10 mm × 10 mm × 2 mm. All Q235 samples were obtained from Shanghai Luosong Electromechanical Equipment Co., Ltd. (Shanghai, China).

**Instrumentation**. An electrochemical workstation (Vertex, Ivium Technologies, Eindhoven, The Netherlands) was used for electrochemical measurements. Surface morphology and three-dimensional roughness analyses were performed using a confocal microscope (Smartproof 5, Carl Zeiss AG, Oberkochen, Germany).

### 3.2. Methods

#### 3.2.1. Electrochemical Testing

##### Preparation of Test Solutions

To better approximate real operating environments, this study investigated the influence of H_2_S on the corrosion inhibition performance of ILs. Accordingly, a simulated corrosive solution was prepared, consisting of 37 wt% HCl, laboratory-prepared H_2_S-saturated aqueous solution, and distilled water, yielding a final composition of 5.87 mM (0.00587 mol L^−1^) H_2_S and 27.43 mM HCl [7,22,23]. The H_2_S-saturated solution was obtained by bubbling hydrogen sulfide gas into distilled water, with concentration adjusted as required. The H_2_S concentration in solution was determined using the standard iodometric method [24]. All distilled water used in experiments was deoxygenated by nitrogen purging prior to preparation.

##### Electrochemical Experiments

Electrochemical impedance spectroscopy (EIS) was employed to evaluate the corrosion inhibition performance of ILs at different concentrations. Measurements were conducted at 303 K, close to ambient temperature [7]. EIS tests were carried out using an Ivium Vertex electrochemical workstation in a conventional three-electrode configuration, with Q235 steel as the working electrode, a platinum wire as the counter electrode, and a saturated calomel electrode (SCE) as the reference. Prior to testing, Q235 working electrodes were polished with sandpaper, immersed in anhydrous ethanol, ultrasonically cleaned, and then dried with filter paper. The three-electrode system was immersed in the test solution for 1200 s to stabilize the open-circuit potential (OCP). Subsequently, EIS measurements were performed at OCP over the frequency range of 100 kHz to 0.1 Hz with a sinusoidal perturbation amplitude of 10 mV. The concentrations of ILs in the test solutions were set to 0, 10, 30, and 50 mM.

#### 3.2.2. Surface Roughness Evaluation of Corroded Specimens

Post-electrochemical specimens were examined using a confocal laser scanning microscope (CLSM). Prior to CLSM measurements, the corroded surfaces were carefully treated to remove corrosion products (e.g., FeS) and residual inhibitors. Specifically, specimens were gently wiped with lint-free cloths moistened with distilled water, followed by nitrogen purging and vacuum drying to avoid secondary oxidation or contamination, thereby minimizing manual artifacts in surface roughness evaluation. During measurements, relatively smooth surface regions were selected, as these areas were more likely to exhibit higher inhibitor film coverage and thus provide representative roughness data reflecting the anti-permeability of IL films. Using the 3D scanning module of the CLSM, pseudo-color surface images were obtained. The accompanying analysis software (Confomap) was then employed to generate three-dimensional topography and cross-sectional profiles of the selected regions, from which the arithmetic mean roughness (S_a_) values of the specimens treated with the three ILs inhibitors were directly extracted.

#### 3.2.3. Evaluation of Anti-Permeability

To establish a framework for assessing the anti-permeability of ILs films, the overall inhibition performance was systematically compared with surface roughness measurements. Two scenarios were distinguished.

In the first scenario, when the overall inhibition efficiency was below 70% and the ranking of surface roughness was consistent with that of inhibition efficiency, the inhibition effect was preliminarily attributed to insufficient surface coverage. In this case, the inhibitor concentration was increased until the efficiency approached a plateau or commenced to decline. If, under these conditions, the roughness ranking continued to follow the inhibition efficiency trend, it was concluded that anti-permeability correlated with overall inhibition performance. However, if the roughness ranking deviated from the efficiency trend upon increasing concentration, the roughness order under these conditions was adopted as the indicator of anti-permeability.

In the second scenario, if the initial experiments showed that the surface roughness ranking was independent of inhibition efficiency, the roughness order obtained in the initial tests was directly used to represent the relative anti-permeability of the inhibitors. In this case, surface roughness was recognized as an effective and independent metric for evaluating anti-permeability.

## 4. Conclusions

This study innovatively proposes a dual-criterion framework that combines inhibition efficiency measured by electrochemical impedance spectroscopy (EIS) with surface roughness (S_a_) obtained from confocal laser scanning microscopy (CLSM) to evaluate the anti-permeability of imidazolium-based ionic liquid corrosion inhibitors in H_2_S/HCl environments. Among the three ionic liquids ([C_3_mim][Br], [C_3_mim][NO_3_], and [C_3_mim][OTf]) in the concentration range of 10–50 mM, the nitrate system consistently exhibited the lowest surface roughness (S_a_ = 12.2 μm at 50 mM) and the best anti-permeability, despite its overall inhibition efficiency (81.27%) being lower than that of the trifluoromethanesulfonate system (91.68%, S_a_ = 14.79 μm). This apparent discrepancy indicates that high coverage does not necessarily guarantee a compact film. The dual-criterion framework established in this study thus provides a universal approach: for systems with inhibition efficiency below 73%, concentration-gradient testing can eliminate coverage-related interference, whereas under high-coverage conditions, anti-permeability should be directly assessed based on surface roughness ranking. In summary, the dual-criterion framework integrates inhibition efficiency and surface roughness, revealing the distinct roles of surface coverage and anti-permeability in ionic liquid corrosion inhibition.

## Figures and Tables

**Table 1:** EIS parameters of Q235 obtained from equivalent circuit fitting (a: [C_3_mim][OTf]; b: [C_3_mim][NO_3_]; c: [C_3_mim][Br]).

*ILs*	*C* (mM)	*R_s_* (Ω cm^2^)	*Y_f_* × 10^−4^ (*S s^n^* cm^−2^)	*n_f_*	*R_f_* (Ω cm^2^)	*Y_o_* × 10^−4^ (*S s^n^* cm^−2^)	*n_o_*	*R_ct_* (Ω cm^2^)	*L* (H cm^2^)	*R_L_* (Ω cm^2^)	*R_p_* (Ω cm^2^)	*η* (%)	*χ*^2^ × 10^−4^
Blank	—	2.033	—	—	—	20	0.826	4.005	0.2231	1.044	5.049	—	18.4
a	10	5.213	34.9	0.9291	3.235	1.95	0.9123	20.24	17.15	3.907	27.382	81.56	1.05
b	10	6.246	35.1	0.8692	3.07	2.18	0.8868	16.79	1.861	2.375	22.235	77.29	0.415
c	10	5.433	35.2	0.8864	2.964	2.37	0.8997	14.15	1.732	2.161	19.275	73.81	0.375
a	30	4.443	9.60	0.9771	10.72	9.93	1	32.72	0.6612	0.7397	44.1797	88.57	7.17
b	30	2.022	1.40	1	3.934	9.19	0.5559	18.74	4.189	2.985	25.659	80.32	2.61
c	30	3.898	19.2	0.8836	3.898	2.00	0.9064	17.71	2.092	2.396	24.004	78.97	1.39
a	50	1.429	1.17	1	50.21	17.9	1	10.47	—	—	60.68	91.68	38
b	50	2.303	1.25	1	15.93	18.5	0.8044	8.231	3.528	2.799	26.96	81.27	0.538
c	50	2.98	13.6	0.8441	8.561	1.48	0.9521	20.44	7.78	4.071	33.072	84.73	2.08

**Table 2:** Arithmetic mean surface roughness (S_a_) values.

Arithmetic Mean Surface Roughness (S_a_) Unit: μm					
[C_3_mim][OTf]-10 mM	15.55	[C_3_mim][OTf]-30 mM	15.32	[C_3_mim][OTf]-50 mM	14.79
[C_3_mim][NO_3_]-10 mM	13.66	[C_3_mim][NO_3_]-30 mM	12.54	[C_3_mim][NO_3_]-50 mM	12.2
[C_3_mim][Br]-10 mM	21.06	[C_3_mim][Br]-30 mM	17.06	[C_3_mim][Br]-50 mM	16.99

## Data Availability

The original contributions presented in this study are included in the article. Further inquiries can be directed to the corresponding author.

## Abbreviations

The following abbreviations are used in this manuscript:

ILs	ionic liquids
[C_3_mim][OTf]	1-propyl-3-methylimidazolium trifluoromethanesulfonate
[C_3_mim][NO_3_]	1-propyl-3-methylimidazolium nitrate
[C_3_mim][Br]	1-propyl-3-methylimidazolium bromide
EIS	Electrochemical impedance spectroscopy
CLSM	confocal laser scanning microscope
SCE	saturated calomel electrode
OCP	open-circuit potential
3D	three-dimensional
